# Response to comment on “Pooled analysis of Xpert bladder cancer based on the 5 mRNAs for rapid diagnosis of bladder carcinoma”

**DOI:** 10.1186/s12957-022-02771-3

**Published:** 2022-09-24

**Authors:** Yu-Fei Xie, Ye-Ling Liu, Xu-Guang Guo

**Affiliations:** 1 Department of Clinical Laboratory Medicine, The Third Affiliated Hospital of Guangzhou Medical University, Guangzhou, 510150 China; 2 Department of Clinical Medicine, The Third Clinical School of Guangzhou Medical University, Guangzhou, 511436 China; 3 Key Laboratory for Major Obstetric Diseases of Guangdong Province, Guangzhou, 510150 China; 4 Key Laboratory of Reproduction and Genetics of Guangdong Higher Education Institutes, Guangzhou, 510150 China; 5 Guangzhou Key Laboratory for Clinical Rapid Diagnosis and Early Warning of Infectious Diseases, KingMed School of Laboratory Medicine, Guangzhou Medical University, Guangzhou, China

**Keywords:** Bladder cancer, NMIBC, Xpert BC

## Abstract

This article is a response to “comment on ‘Pooled analysis of Xpert bladder cancer based on the 5 mRNAs for rapid diagnosis of bladder carcinoma’” by Gopal Sharma. With this comment, author highlighted the strengths and limitations of the study. With this response, we respond to this and make a comparison of the results and conclusions.

We thank Gopal Sharma for his interest and insightful comments [1] on our published work [2]. The article seeks to make a pooled analysis of Xpert bladder cancer (Xpert BC) based on the five mRNAs for rapid diagnosis of bladder carcinoma, compared with cystoscopy and/or biopsy. In terms of clinical application prospects, Xpert BC is supposed to be used for tumor recurrences and the screening of patients with hematuria. Therefore, samples of hematuria patients were included in this study. However, due to the small number of hematuria patients, the study mainly included the patients with bladder cancer on follow-up. Consequently, the conclusion is more suitable for the follow-up of bladder cancer patients. We agree that Gopal Sharma’s suggestions are reasonable and find that Sharma et al. also pool the data regarding the diagnostic performance of Xpert BC in patients with non-muscle invasive bladder cancer (NMIBC) [3]. The data of hematuria patients in the studies of Valenberg et al. [4] and Wallace et al. [5] were excluded, and 5 more articles from the updated literature search were added by Sharma et al.

The pooled sensitivity and specificity of Xpert BC in the diagnosis of bladder cancer were 0.71 and 0.81 in our research, which is little different from the sensitivity (0.73) and specificity (0.77) of Sharma et al. All articles used cystoscopy and/or biopsy as the gold standard, and the included studies did not get enough data for subgroup analysis of the gold standard, which is consistent with Sharma et al. Besides, our conclusions on the diagnostic performance of Xpert BC in follow-up patients are consistent with Sharma et al.; Xpert BC has acceptable diagnostic accuracy in monitoring patients with NMIBC. And the diagnostic accuracy of high-grade tumors was higher than that of low-grade tumors in subgroup analysis.

Our research still has limitations caused by factors such as heterogeneity. More studies in the future are supposed to contribute to subgroup analysis and heterogeneity resolution.

## Data Availability

All data analyzed during this study are included in this published article.

## Abbreviations

Xpert BC: Xpert bladder cancer
NMIBC: Non-muscle-invasive bladder cancer
